# A randomized controlled trial of trans-intersphincteric double seton (TRISDS) for the treatment of perianal abscess

**DOI:** 10.1093/gastro/goaf091

**Published:** 2025-09-30

**Authors:** Leichang Zhang, Pan Shen, Xiao Yuan, Guanghua Chen, Wei Ge, Wu Liao, Xiaonan Zhang, Chen Wang, Lu Li

**Affiliations:** Department of Anorectal Surgery, Affiliated Hospital of Jiangxi University of Chinese Medicine, Nanchang, Jiangxi, P. R. China; Clinical Medical College, Jiangxi University of Chinese Medicine, Nanchang, Jiangxi, P. R. China; Clinical Medical College, Jiangxi University of Chinese Medicine, Nanchang, Jiangxi, P. R. China; Clinical Medical College, Jiangxi University of Chinese Medicine, Nanchang, Jiangxi, P. R. China; Department of Anorectal Surgery, Affiliated Hospital of Jiangxi University of Chinese Medicine, Nanchang, Jiangxi, P. R. China; Clinical Medical College, Jiangxi University of Chinese Medicine, Nanchang, Jiangxi, P. R. China; Department of Anorectal Surgery, Affiliated Hospital of Jiangxi University of Chinese Medicine, Nanchang, Jiangxi, P. R. China; Clinical Medical College, Jiangxi University of Chinese Medicine, Nanchang, Jiangxi, P. R. China; Department of Anorectal Surgery, Affiliated Hospital of Jiangxi University of Chinese Medicine, Nanchang, Jiangxi, P. R. China; Clinical Medical College, Jiangxi University of Chinese Medicine, Nanchang, Jiangxi, P. R. China; Clinical Medical College, Jiangxi University of Chinese Medicine, Nanchang, Jiangxi, P. R. China; Department of Anorectal Surgery, Longhua Hospital, Shanghai University of Traditional Chinese Medicine, Shanghai, P. R. China; Department of Anorectal Surgery, Affiliated Hospital of Jiangxi University of Chinese Medicine, Nanchang, Jiangxi, P. R. China; Clinical Medical College, Jiangxi University of Chinese Medicine, Nanchang, Jiangxi, P. R. China

**Keywords:** trans-intersphincteric double seton, TRISDS, perianal abscess, anal fistula, incision and drainage, anorectal

## Abstract

**Background:**

Incision and drainage (I&D) for perianal abscesses is associated with high rates of fistula formation. Our study aimed to evaluate the effectiveness of a novel technique, trans-intersphincteric double seton (TRISDS), designed to preserve anal sphincter integrity and improve clinical outcomes compared to I&D.

**Methods:**

This prospective, randomized, non-blinded controlled study included adult patients with perianal abscesses located below the levator ani muscle with an internal opening. Patients were randomly assigned to either the TRISDS group (*n *= 55) or the I&D group (*n *= 51). The TRISDS technique involved two incisions: intersphincteric and drainage incisions with the placement of two loose setons. One seton was positioned to preserve the internal anal sphincter and facilitate drainage through the intersphincteric space, while the other seton aimed to protect the external anal sphincter to ensure comprehensive drainage. The I&D group underwent conventional I&D without damaging the anal sphincter complex. The primary outcome was the cure rate of perianal abscesses, which was defined as complete epithelialization of wounds without fistula or exudate and no recurrence within 12 months after surgery.

**Results:**

The TRISDS group achieved a significantly higher cure rate of 78.2% (43/55) compared to 41.2% (21/51) in the I&D group (*P *< 0.05). There were no significant differences in anal function at 2 months postoperatively between the groups (median Wexner score, IQR: 1.0 [0.0–1.0] vs 1.0 [0.0–1.0], *P *> 0.05).

**Conclusions:**

The study highlighted the effectiveness of TRISDS in improving cure rate without compromising anal function. The TRISDS technique represents a promising strategy for the treatment of perianal abscesses. Further multicenter studies are recommended to validate these findings and expand the application of TRISDS in diverse patient populations.

## Introduction

Perianal abscess (PA) is an acute suppurative infection. Approximately 90% of idiopathic PAs stem from infections in the anal glands [1, 2], predominantly developing in the perianal sphincter space surrounding these glands [3].

Once diagnosed, prompt surgical intervention is recommended by guidelines. The American Society of Colon and Rectal Surgeons (ASCRS) recommends prompt incision and drainage (I&D) to ensure adequate drainage while optimally preserving sphincter function [4]. Despite being the standard treatment, I&D is associated with a high incidence of fistula formation or abscess recurrence [5]. Specifically, ischiorectal abscesses and intersphincteric abscesses have a significantly higher likelihood of fistula formation following simple drainage. Sözener *et al.* [6] demonstrated that, in patients undergoing only anorectal abscess drainage, the risk of fistula formation increased 7.82-fold for ischiorectal abscesses and 3.07-fold for intersphincteric abscesses, compared with PAs. In contrast, abscess drainage combined with fistulotomy performed in a single session has shown greater effectiveness in treating PAs by simultaneously reducing recurrence and fistula formation rates. However, this combined approach may carry a higher risk of postoperative sphincter dysfunction, potentially leading to an increased risk of fecal incontinence (FI) [7–11].

To effectively treat PAs while preserving anal function, a novel approach, the trans-intersphincteric double seton (TRISDS) technique, has been developed. This method is based on the modified Parks loose seton technique [12]. This innovative trans-intersphincteric approach is engineered to preserve the anatomical integrity of the entire sphincter complex. It employs two distinct setons: one is placed to protect the internal anal sphincter (IAS) through an intersphincteric approach, and the other is placed to protect the external anal sphincter (EAS). The TRISDS is designed to provide adequate drainage of the infection focus. Crucially, since the internal and external sphincters are separated rather than incised, the risk of postoperative FI may be reduced. The TRISDS technique, developed to address the limitations of conventional I&D procedures, has attracted growing attention in the surgical community. Its detailed methodology and initial results were presented at the 2024 ASCRS Annual Meeting, reflecting its potential impact on the clinical management of PAs.

It was hypothesized that TRISDS, by effectively addressing the primary infection focus with setons while preserving both the IAS and EAS, may result in lower rates of postoperative recurrence, fistula formation, and FI compared to conventional I&D. However, it is anticipated that the multiple incisions and the presence of seton placement required by TRISDS might lead to more severe postoperative pain and edema, potentially leading to longer healing time. Therefore, this study aims to compare the efficacy and safety of TRISDS versus I&D in treating PAs by evaluating fistula formation rates, postoperative pain, healing time, and anal function scores. Through clarifying these outcomes, the study is intended to enrich the therapeutic landscape and guide clinical decision-making for this condition.

## Methods

### Study design and ethical statement

A randomized parallel-controlled, non-blind prospective clinical trial was designed. The study enrolled participants with PAs treated between September 2020 and September 2023 in the department of anorectal surgery at the Affiliated Hospital of Jiangxi University of Chinese Medicine. This protocol was approved by the Chinese Clinical Trial Registry (ChiCTR2000032941) and the Medical Ethics Committee of the Affiliated Hospital of Jiangxi University of Chinese Medicine (JZFYKYLL20200420007). Written informed consent was obtained from all patients.

### Eligibility criteria

The study included patients aged 18–65 years with PAs diagnosed via clinical manifestations and magnetic resonance imaging as being located below the levator ani muscle, without evidence of muscle penetration, and with an identifiable internal opening. The exclusion criteria were as follows: (i) patients with high PAs located at or above the levator ani muscle; (ii) absence of an internal fistula opening; (iii) suspicion of Fournier’s gangrene; (iv) recognized other infection at the time of surgery; (v) confirmed inflammatory bowel disease; (vi) secondary and recurrent PAs; (vii) pre-existing symptoms of FI; (viii) history of related treatments; (ix) any additional surgical procedure performed; (x) individuals who were receiving immunosuppressive or anticoagulant therapy; (xi) diagnosed with diabetes mellitus; (xii) known chronic disabling diseases; and (xiii) who were pregnant or lactating.

### Preoperative assessment

All patients had a detailed medical history recorded, including chief complaints, symptoms, duration, past trauma and surgical history, current comorbidities and ongoing treatment plans, degree of anal pain, and bowel movement patterns.

Examinations were conducted with patients in the knee–chest position, and the location of the abscess cavity was determined through observation, digital rectal examination, and anoscopy while excluding other anorectal diseases. The preoperative anal function was quantitatively assessed using the Wexner score. To ensure standardized inclusion criteria, all patients underwent perianal magnetic resonance imaging to confirm the diagnosis and determine the extent of the abscess cavity.

### Random sequence generation and blinding

Patients enrolled in the study were randomized into two intervention groups by an independent statistician not involved in the trial. Group 1 received double seton placement via an intersphincteric approach to protect the IAS and EAS. Group 2 underwent adequate I&D without damaging the anal sphincter complex.

Randomization was performed using online randomization software (www.randomization.com), with allocation concealment achieved through the sealed envelope method. Due to practical considerations, blinding the surgical operator was not feasible. Similarly, patients were not blinded to uphold their right to be informed about the procedure; thus, patients were aware of whether they received seton placement. Additionally, blinding of follow-up team was not feasible, as they could easily distinguish between a single incision (I&D group) and multiple incisions (seton group).

### Surgical interventions

All patients participating in the study provided informed consent and signed consent forms. They were informed about the principles, potential benefits, recurrence rates, and possible complications of each procedure.

Patients were placed in the lateral position and underwent surgery under regional anesthesia with ropivacaine. Specifically, the choice of side was determined by the PA location: left lateral position was used for left-sided PA, right lateral position for right-sided PA, and right lateral position for anterior or posterior midline PA. Three senior colorectal surgeons performed all surgeries.

**Figure 1.**
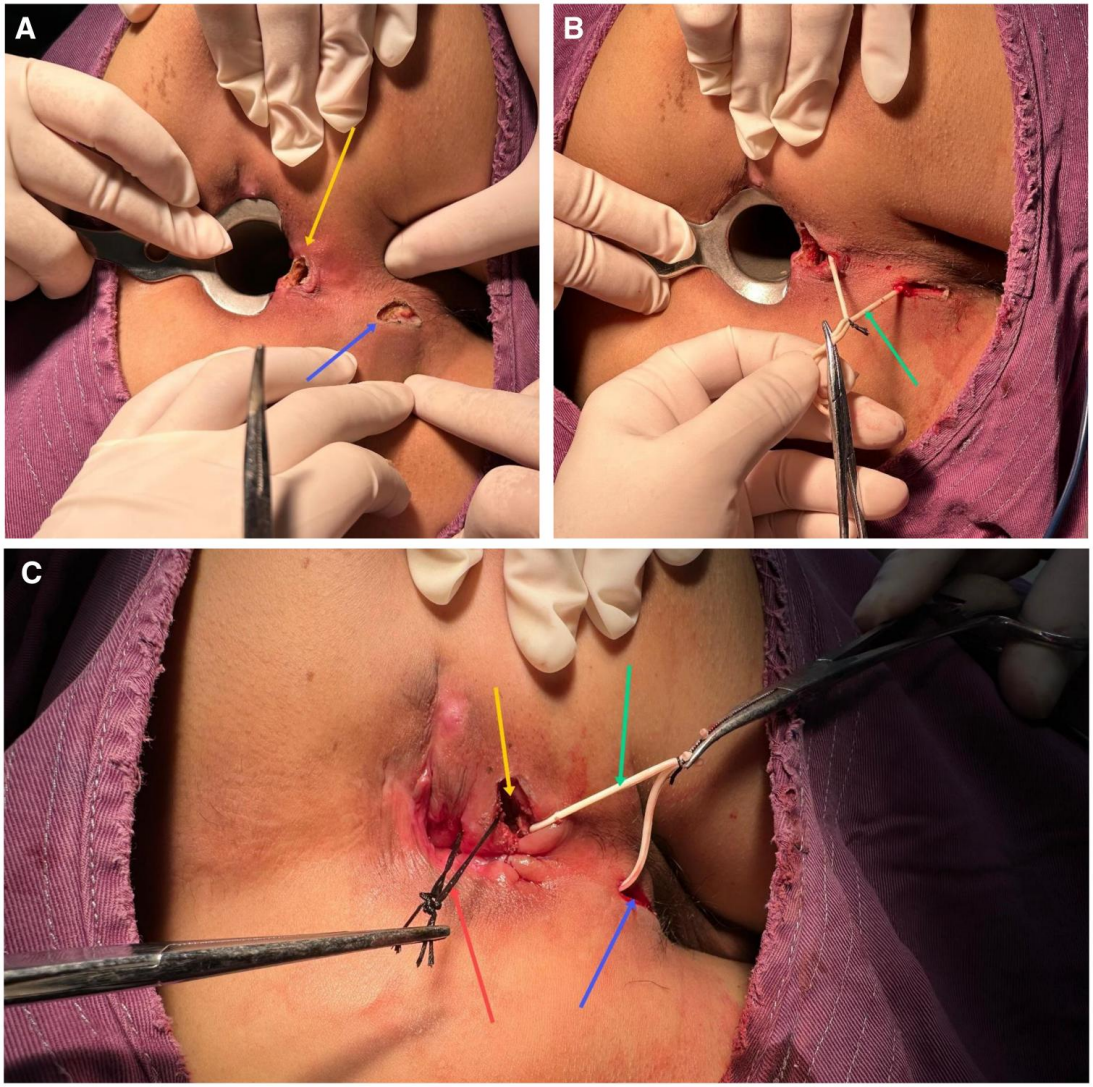
Surgical procedure of the TRISDS technique. (A) An intersphincteric incision and a drainage incision were made. (B) The first seton was placed, and the external anal sphincter was not incised. (C) The second seton was placed, and the internal opening was managed. Yellow arrow: the intersphincteric incision between the internal anal sphincter and external anal sphincter; blue arrow: the drainage incision is located at the edge of the abscess; green arrow: loose seton from the intersphincteric incision to the drainage incision; red arrow: loose seton from the intersphincteric incision to the internal opening. TRISDS = trans-intersphincteric double seton.

Group 1 (TRISDS group): an intersphincteric incision was made between the IAS and EAS. As the incision deepened, the IAS and EAS muscles were gradually separated until reaching the internal opening. Subsequently, a drainage incision was made at the site of the abscess, while preserving the EAS. The surgeon identified a track from the drainage incision into the intersphincteric incision. Two setons (either silicone or silk) were prepared. The first seton was placed along this track between the two incisions. The second seton was positioned between the intersphincteric incision and the internal opening (Figure 1). Detailed surgical procedures are available in the presentation “VM51—Trans-intersphincteric Double Seton for Perianal Abscess” at the 2024 ASCRS Annual Scientific Meeting [13]. The seton at the internal sphincter was removed after 3 days, while the seton at the external sphincter was removed after 7 days. The timing of seton removal was based on anatomical and clinical considerations. The intersphincteric space between the internal and external sphincters is relatively shallow, allowing for early removal of the internal seton. In contrast, the abscess cavity associated with the external sphincter is usually broader, thus requiring a longer drainage period before removing the external seton. In clinical practice, seton removal is often determined flexibly based on the tightness and drainage status of the seton. However, to ensure protocol consistency and minimize bias in this controlled study, we adopted a standardized schedule for seton removal.

Group 2 (I&D group): a radial incision was made at the most prominent site of abscess fluctuation to drain the pus. The incision was expanded as much as possible to ensure adequate drainage without damaging the anal sphincter complex.

### Postoperative assessment and follow-up

Follow-ups were conducted at 24 h, 1 week, 2 weeks, 2 months, and 12 months postoperatively. Patients were transferred to the inpatient department immediately after surgery. During hospitalization, follow-ups (24 h) were conducted in the inpatient ward, and the length of hospital stay was recorded. After discharge, subsequent follow-ups (1 week, 2 weeks, 2 months, and 12 months) were carried out in the outpatient department. These follow-ups were conducted by a surgical resident and an attending physician. Each follow-up recorded healing progress, pain levels, complications, and anal function scores. Pain was assessed using a visual analogue scale (VAS) at 24 h, 1 week, and 2 weeks postoperatively. Wound exudation was evaluated on a scale of 0–3, with 0 indicating no secretion, 1 indicating secretion not penetrating one layer of gauze, 2 indicating secretion penetrating one layer of gauze but not the second, and 3 indicating secretion penetrating two or more layers of gauze. Granulation growth was also assessed on a scale of 0–3, with 0 indicating good granulation growth, 1 indicating bright red granulation that bleeds easily when rubbed, 2 indicating light red granulation that does not bleed easily when rubbed, and 3 indicating less granulation with light grayish–white granulation that does not bleed easily when rubbed. Edema was evaluated on a scale of 0–3, with 0 indicating no edema at the trauma edge, 1 indicating slight edema at the trauma edge, 2 indicating redness and swelling that slightly affects fecal evacuation, and 3 indicating extensive edema at the trauma edge that seriously affects fecal evacuation and daily life. Anal function was assessed using the Wexner score (range: 0–20, with higher scores indicating more severe FI) at preoperatively, at 2 weeks postoperatively, and at 2 months postoperatively. For recurrent abscesses and fistulas, drainage and fistulotomy were performed as needed.

### Outcomes of the study

The primary outcome was the cure rate of PAs, defined as complete wound healing (complete epithelialization with no fistula or exudate) and no recurrence within 12 months after surgery [14].

Secondary outcomes included anal sphincter function (measured by the median Wexner score), length of hospital stay, postoperative pain, and wound healing characteristics (wound exudate, bud growth, and traumatic edema).

### Sample size calculation

The required sample size for this study was calculated based on the primary outcome of the cure rate for each group. Literature analysis revealed no similar clinical trials analyzing seton treatment for PAs. However, the three-cavity clearance (TCC) method, which separates the IAS and EAS and achieves sufficient drainage in the submucosal, intersphincteric, and extrasphincteric spaces [15], serves as a reference due to its similar drainage effectiveness to TRISDS. Based on a randomized trial comparing TCC with I&D, fistula average rates of 13% for TRISDS and 39% for I&D were hypothesized. Using an online sample size calculator (https://clincalc.com/Stats/SampleSize.aspx), with α set at 5% and study power at 80%, it was determined that each group would require at least 43 patients, resulting in a total of 86 patients for both groups. Considering potential differences between TRISDS and TCC, as well as potential loss to follow-up and dropouts, the study was designed to include 100 patients.

### Statistical analysis

The study primarily based its analyses on the modified intention-to-treat (ITT) set. The modified ITT population was defined as all subjects who actually received the assigned surgical procedure (TRISDS or I&D), since those who did not receive the allocated treatment were not expected to benefit from the procedure and were therefore excluded from the primary analysis set. To assess the stability of the primary outcome in the presence of missing data, we conducted an additional sensitivity analysis. This analysis incorporated all subjects with missing follow-up data and constructed two theoretical extreme scenarios. In the best-case scenario, it was assumed that all missing subjects in the TRISDS group were cured with a Wexner score equal to the minimum observed value, while all missing subjects in the I&D group were considered uncured with a Wexner score equal to the maximum observed value—thus providing the most favorable assessment for TRISDS efficacy. Conversely, in the worst-case scenario, it was assumed that all missing subjects in the TRISDS group remained uncured with a Wexner score equal to the maximum observed value, whereas all missing subjects in the I&D group were deemed cured with a Wexner score equal to the minimum observed value, representing the most unfavorable conditions for TRISDS. By comparing two extreme scenarios, we assessed whether our study’s conclusions retained sufficient robustness and credibility in the presence of potential attrition bias.

Results were analyzed using SPSS 26.0 statistical software. For continuous variables, data were tested for normality using the Shapiro–Wilk test. Normally distributed variables were expressed as mean ± standard deviation (SD) and compared using the independent samples *t*-test. Non-normally distributed or ordinal variables, such as Wexner scores and wound condition, were expressed as median (interquartile range [IQR]) and compared using the Mann–Whitney *U* test. Categorical variables, including cure rates and the incidence of fistula formation or abscess recurrence, were expressed as counts and percentages and compared using the chi-square (χ^2^) test. A two-sided *P*-value <0.05 was considered statistically significant.

## Results

### Patient enrollment and baseline characteristics

**Figure 2.**
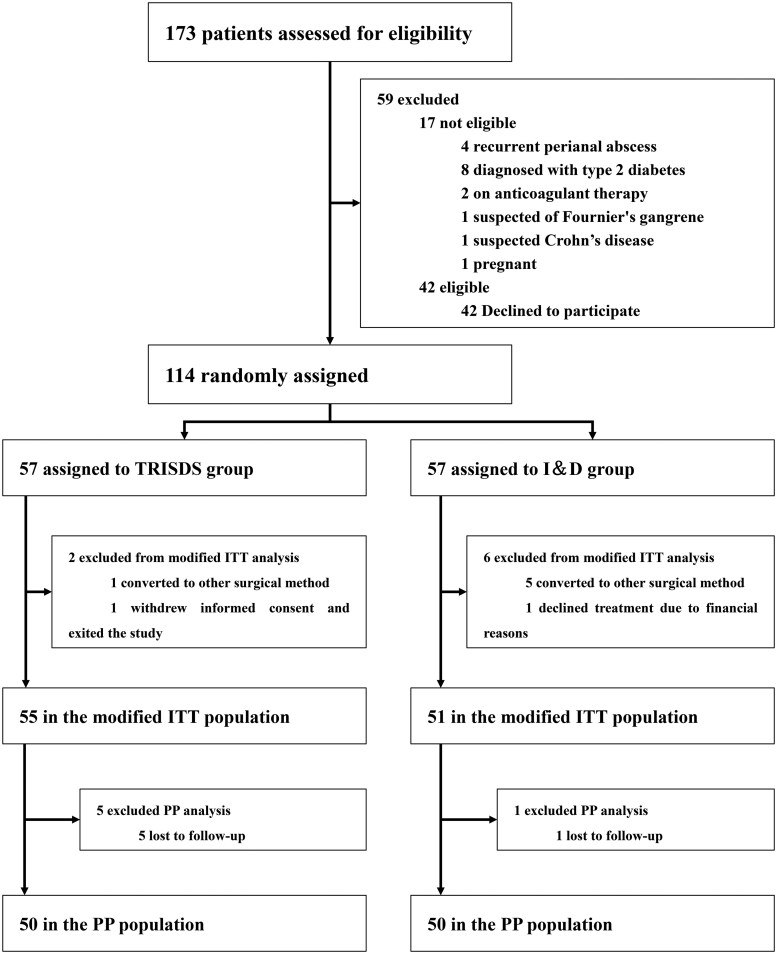
CONSORT (Consolidated Standards of Reporting Trials) flow chart illustrating the process of patient selection and exclusion. TRISDS = trans-intersphincteric double seton, I&D = incision and drainage, ITT = intention-to-treat, PP = per-protocol.

Following the initial evaluation, 106 out of 173 patients with PAs were analyzed in this randomized clinical trial. Participants were recruited as scheduled, and recruitment was terminated once the target number was reached. The allocation and exclusion process is illustrated in the CONSORT flow diagram (Figure 2). The study comprised 83 males (78.3%) and 23 females (21.7%), with an average age of 33.06 years. Patients in Group 1 were treated with the TRISDS method, while those in Group 2 underwent I&D. As illustrated in Table 1, there were no significant differences in age, sex, or clinical presentation between the two groups.

**Table 1.** Comparison of baseline data between TRISDS and I&D groups

Variable	TRISDS (*n *= 55)	I&D (*n *= 51)	*P*
Age (years), mean ± SD	33.27 ± 9.97	32.82 ± 8.89	0.808
Male, *n* (%)	44 (80.0）	39 (76.5)	0.660
Duration of symptoms, days, mean ± SD	5.27 ± 1.51	5.52 ± 2.38	0.512
Loss to follow-up, *n* (%)	5 (9.1)	1 (2.0)	0.243
Body mass index, mean ± SD	22.62 ± 2.50	23.22 ± 3.13	0.281

TRISDS = trans-intersphincteric double seton, I&D = incision and drainage, SD = standard deviation.

### Main outcome

The TRISDS group exhibited a significantly higher cure rate (78.2%, 43/55, 95% confidence interval (CI): 65.7%–87.0%) than the I&D group (41.2%, 21/51, 95% CI: 28.7%–54.9%; *P* < 0.001). Fistula formation occurred in 9.1% of TRISDS patients vs 35.3% in the I&D group (*P* = 0.001), and recurrence of abscesses was also significantly lower in the TRISDS group (3.6% vs 21.6%, *P* = 0.005). In addition, there was no significant difference in anal function at 2 months postoperatively, with median Wexner scores [IQR] of 1.0 [0.0–1.0] in the TRISDS group and 1.0 [0.0–1.0] in the I&D group (*P* = 0.290). All these results were derived from the modified ITT analysis. These results suggest that TRISDS was associated with improved clinical outcomes without compromising anal sphincter function.

### Wound condition

Table 2 presents the surgical wound outcomes at 24 h, 1 week, and 2 weeks postoperatively for both groups. The results indicated no significant differences in wound exudate, bud growth, or traumatic edema between the two groups (*P *> 0.05).

**Table 2.** Comparison of conditions of wound between TRISDS and I&D groups

Variable	TRISDS (*n *= 55)	I&D (*n *= 51)	*P*
Wound exudate, median (IQR)			
Postoperative 24 h	3.0 (2.0–3.0)	3.0 (2.0–3.0)	0.097
Postoperative 1 week	2.0 (1.0–2.25)	2.0 (1.0–2.0)	0.296
Postoperative 2 weeks	1.0 (0.0–1.0)	1.0 (0.0–1.0)	0.275
Bud growth, median (IQR)			
Postoperative 24 h	3.0 (2.0–3.0)	3.0 (2.0–3.0)	0.164
Postoperative 1 week	2.0 (1.0–2.0)	2.0 (1.0–2.0)	0.129
Postoperative 2 weeks	1.0 (0.25–1.75)	1.0 (1.0–2.0)	0.192
Traumatic edema, median (IQR)			
Postoperative 24 h	0.0 (0.0–1.0)	0.0 (0.0–1.0)	0.482
Postoperative 1 week	0.0 (0.0–1.0)	1.0 (0.0–1.0)	0.576
Postoperative 2 weeks	0.0 (0.0–1.0)	0.0 (0.0–1.0)	0.453

TRISDS = trans-intersphincteric double seton, I&D = incision and drainage, IQR = interquartile range.

### Postoperative pain

At 24 h after surgery, the VAS pain score was 7.40 ± 1.01 for the TRISDS group and 6.86 ± 1.04 for the I&D group (*P *= 0.008). However, no significant differences in VAS pain scores were observed at the 1- and 2-week follow-ups (3.59 ± 1.14 vs 3.37 ± 0.98, *P *= 0.293; 1.35 ± 0.59 vs 1.58 ± 0.91, *P *= 0.128) (Table 3).

**Table 3.** Comparison of outcome between TRISDS and I&D groups

Variable	TRISDS (*n *= 55)	I&D *n *= 51)	*P*
Pain score, mean ± SD			
Postoperative 24 h	7.40 ± 1.01	6.86 ± 1.04	0.008*
Postoperative 1 week	3.59 ± 1.14	3.37 ± 0.98	0.293
Postoperative 2 weeks	1.35 ± 0.59	1.58 ± 0.91	0.128
Length of hospital stay, days, mean ± SD	3.96 ± 1.51	3.37 ± 1.41	0.041*
Treatment failure, *n* (%)			
Fistula formation	5 (9.1)	18 (35.3)	0.001*
Recurrence of abscesses	2 (3.6)	11 (21.6)	0.005*
Total	7 (12.7)	29 (56.9)	<0.001*
Wexner score, median (IQR)			
Preoperative	0.0 (0.0–1.0)	0.0 (0.0–1.0)	0.643
Postoperative 2 weeks	1.5 (1.0–2.75)	1.0 (0.0–2.0)	0.020*
Postoperative 2 months	1.0 (0.0–1.0)	1.0 (0.0–1.0)	0.290

TRISDS = trans-intersphincteric double seton, I&D = incision and drainage, SD = standard deviation, IQR = interquartile range.

* *P* < 0.05.

### Hospitalization and recurrence

The hospitalization time in the TRISDS group was 3.96 ± 1.51 days, which was significantly longer (*P *= 0.041) than 3.37 ± 1.41 days in the I&D group (Table 3). Clinical outcomes revealed a significant difference in cure rates between the two groups. The TRISDS group exhibited a 78.2% cure rate (43/55), significantly higher than the 41.2% cure rate (21/51) observed in the I&D group. While both groups experienced some treatment failures, the TRISDS group had fewer treatment failures (7 patients: 5 developed fistulas and 2 had recurrent abscesses) compared to the I&D group (29 patients: 18 developed fistulas and 11 had recurrent abscesses) (*P *< 0.001).

### Anal function

**Figure 3.**
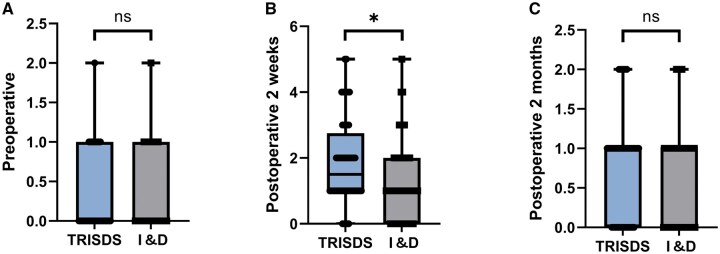
Comparison of Wexner scores (median and interquartile range) between TRISDS and I&D groups preoperatively, at 2 weeks postoperatively, and at 2 months postoperatively. Statistical comparisons were performed using the Mann–Whitney *U* test. ns, *P* > 0.05; *P < 0.05. (A) Preoperative Wexner scores showed no significant differences between groups. (B) At 2 weeks postoperatively, the TRISDS group exhibited significantly higher Wexner scores compared with the I&D group. (C) At 2 months postoperatively, there were no significant differences in Wexner scores between groups. TRISDS = trans-intersphincteric double seton, I&D = incision and drainage, ns = non-significant.

There were no significant differences in preoperative Wexner scores between the two groups (*P *> 0.05). At 2 weeks postoperatively, the TRISDS group exhibited significantly higher Wexner scores than the I&D group (median Wexner score [IQR]: 1.5 [1.0–2.75] vs 1.0 [0.0–2.0], *P *= 0.020), indicating a transient difference during the early healing phase. However, there were no significant differences between the two groups at 2 months after surgery (median Wexner score [IQR]: 1.0 [0.0–1.0] vs 1.0 [0.0–1.0], *P *= 0.290). No patients in either group experienced FI during the follow-up period (Figure 3 and Table 3).

### Sensitivity analysis

To assess the potential impact of missing data on the primary outcome, we performed a sensitivity analysis that included all six patients lost to follow-up (five from the TRISDS group and one from the I&D group).

Under the best-case scenario, we assumed that all missing subjects in the TRISDS group were cured with a Wexner score of 0 (the lowest observed value), while those in the I&D group were assumed to remain uncured with a Wexner score of 2 (the highest observed value). In contrast, under the worst-case scenario, we assumed that all missing subjects in the TRISDS group were uncured with a Wexner score of 2 (the highest observed value), whereas those in the I&D group were considered cured with a Wexner score of 0 (the lowest observed value). Sensitivity analysis indicated that, under both best-case and worst-case scenario assumptions, the conclusions regarding the primary outcome remained unchanged (Table 4).

**Table 4.** Sensitivity analysis of primary outcomes under different imputation scenarios and per-protocol analysis

Variable	TRISDS	I&D	*P*
Sensitivity analysis: best-case scenario			
Cure, *n/n* (%)	48/55 (87.3)	21/51 (41.2)	<0.001*
Wexner score (postoperatively 2 months), median (IQR)	1.0 (0.0–1.0)	1.0 (0.0–1.0)	0.739
Sensitivity analysis: worst-case scenario			
Cure, *n/n* (%)	43/55 (78.2)	22/51 (43.1)	<0.001*
Wexner score (postoperatively 2 months), median (IQR)	1.0 (0.0–1.0)	1.0 (0.0–1.0)	0.063
Per-protocol analysis			
Cure, *n/n* (%)	43/50 (86.0)	21/50 (42.0)	<0.001*
Wexner score (postoperatively 2 months), median (IQR)	1.0 (0.0–1.0)	1.0 (0.0–1.0)	0.290

TRISDS = trans-intersphincteric double seton, I&D = incision and drainage, IQR = interquartile range.

* *P* < 0.05.

In addition, to evaluate the consistency of treatment effects across analysis populations, we compared the results of the per-protocol and modified ITT analyses. The findings demonstrated no significant differences between the two groups for either outcome, thereby supporting the robustness of the treatment effect and the reliability of our conclusions. Detailed per-protocol analysis results are provided in Supplementary Tables 1–3.

## Discussion

PA is a prevalent infectious condition requiring surgical intervention, as conservative management is typically ineffective. The current mainstream surgical approach is I&D, but this procedure is hampered by a high rate of subsequent anal fistula formation reported in 9%–66% of cases. To mitigate this risk, some clinicians perform primary fistulotomy, which involves resecting suspected fistulas during abscess drainage. However, during the acute abscess stage, surrounding tissues are severely inflamed and edematous, complicating the identification of the internal orifice. Blind exploration might create false tracts, causing further tissue damage and increasing the risk of postoperative anal defects or FI [16]. The randomized controlled trial by Schouten and van Vroonhoven revealed a significant likelihood of postoperative sphincter dysfunction following primary fistulectomy and partial internal sphincterotomy [17]. Additionally, a meta-analysis indicated an elevated incidence of FI due to sphincterotomy [8].

The “glandular theory of infection” suggests that over 90% of PAs result from anal gland infections [1]. The primary infection typically occurs in the intersphincteric space and can extend to other areas, necessitating thorough drainage to prevent fistula formation. Therefore, effective treatment hinges on eliminating the primary infection site and ensuring comprehensive drainage. To address these challenges and optimize anal function preservation, the TRISDS technique was developed. As an innovation based on the modified Parks loose-hanging string technique, TRISDS aims to treat PAs by leveraging their underlying pathogenic mechanisms.

Our study demonstrated that TRISDS was associated with a reduced risk of fistula formation compared with I&D, with a fistula incidence of only 9.1% of TRISDS patients vs 35.3% in the I&D group. TRISDS involves hanging the inner sphincter seton from the intersphincteric approach, thereby addressing the intermuscular primary infection foci and facilitating drainage through friction. This approach potentially eliminates infected anal gland ducts and lowers fistula rates. Furthermore, by maintaining sphincter integrity through the use of an additional seton at the EAS level, TRISDS is intended to facilitate comprehensive drainage.

The findings of this study indicate that TRISDS was significantly effective in treating PAs while preserving anal function, despite the occurrence of anal fistula in some patients after surgery. We hypothesize that the removal of the TRISDS seton on the seventh postoperative day may be a contributing factor. Early removal could lead to incomplete drainage, potentially resulting in superficial healing and subsequent fistula formation. To optimize clinical outcomes, a gradual seton removal approach tailored to individual healing progress was suggested to ensure complete drainage and enhance healing rates.

Importantly, TRISDS did not increase functional impairment of the anal sphincter. The seton passes through the intermuscular groove between the IAS and EAS, causing minimal functional damage compared with more extensive sphincterotomy procedures. The anal function was evaluated 2 weeks postoperatively, when tissue trauma was still resolving, and a statistically significant difference was observed in Wexner scores between the I&D and TRISDS groups (*P *< 0.05). This early discrepancy might be attributed to the presence of the hanging seton and incomplete wound healing, resulting in a defect at the surgical site and contributing to minor FI. By 2 months after surgery, when the wound had healed, no significant differences in anal function were observed, suggesting that TRISDS may help preserve anal function by supporting healing without compromising the integrity of the anal sphincter.

From a clinical standpoint, TRISDS offers a viable alternative to I&D, particularly in cases where preservation of the anal sphincter is paramount. Although the procedure is inherently more complex and entails a marginally longer postoperative hospital stay, it significantly lowers both the incidence of postoperative anal fistula formation and recurrence rates, thereby reducing the need for secondary surgeries, lowering healthcare costs, and ultimately enhancing patient satisfaction and quality of life. Integrating our study findings with clinical experience, the TRISDS technique demonstrates favorable efficacy in patients with ischiorectal or intersphincteric abscesses that present with a clearly defined internal opening. Moreover, for individuals at high risk of sphincter injury—such as females [18], those with anterior abscesses [19], or patients exhibiting mild preoperative incontinence—the sphincter-sparing advantages of TRISDS make it a particularly suitable option. While this approach helps mitigate the risk of recurrence and subsequent reoperations for immunocompromised patients, its benefits may be comparatively less apparent in simple PAs without a definitive internal opening.

This study has several limitations to consider. As a single-center, non-blinded investigation with a relatively small sample size, its generalizability may be limited. The limited sample size also precluded direct comparison of TRISDS with fistulotomy surgery. Additionally, the nature of the surgical interventions made blinding of surgeons and patients infeasible, introducing a potential for bias. Consequently, larger multicenter studies with increased patient numbers, extended follow-up periods, and a variety of surgical techniques are necessary to confirm the positive outcomes observed in this trial.

This study focused primarily on ischiorectal and intersphincteric abscesses, but TRISDS has potential applications beyond these cases. For instance, in the case of high abscesses extending into the supralevator space without breaching the EAS, a seton can be used to hook the IAS, thereby opening the intermuscular pathway and preventing pus retention. In cases of sciorectal abscesses with unclear internal openings, using a seton to hook the EAS can prevent the formation of medically induced pseudo-endopenings. Thus, TRISDS may be adaptable to other types of abscesses and anal fistulas, such as horseshoe abscesses and those associated with Crohn’s disease. In horseshoe abscesses, the seton configuration of TRISDS facilitates comprehensive drainage of bilateral abscess cavities while obviating the need for sphincter incision. In patients with Crohn’s disease, TRISDS, as a relatively mild yet effective drainage method, holds promise in reducing the risk of postoperative FI resulting from repeated tissue resections. Nevertheless, these promising applications require rigorous validation through dedicated multicenter studies before widespread adoption.

## Conclusions

TRISDS was associated with improved clinical outcomes compared with conventional I&D. The TRISDS group experienced significantly lower pain in the early postoperative period. Although treatment failure occurred in both groups, the TRISDS group had a significantly lower rate of fistula formation and abscess recurrence. Postoperative complications and the impact on patients’ lifestyles were comparable between the two groups. These findings suggest that TRISDS may be an effective and safe alternative for treating PAs, offering potential advantages in preserving sphincter integrity and reducing the risk of fistula formation. Further multicenter studies with larger sample sizes and extended follow-up periods are warranted to validate these results and expand the applicability of TRISDS to other types of abscesses and anal fistulas.

## Supplementary Material

goaf091_Supplementary_Data
