# Supplementary material for: A randomized controlled trial of trans-intersphincteric double seton (TRISDS) for the treatment of perianal abscess
Source: Gastroenterol Rep (Oxf). 2025 Sep 30;13:goaf091. doi: 10.1093/gastro/goaf091 (PMC12479394; doi:10.1093/gastro/goaf091)
Supplement: goaf091_Supplementary_Data [file goaf091_supplementary_data.docx]

**Supplementary Table 1.** Comparison of baseline data between the two groups in per-protocol population

| Variable | TRISDS  (*n*=50) | I&D  (*n*=50) | *P* value |
| --- | --- | --- | --- |
| Age, years, mean ± SD | 32.98 ± 9.71 | 32.76 ± 8.97 | 0.907 |
| Male, *n* (%) | 41 (82) | 38 (76) | 0.461 |
| Duration of symptoms, days, mean ± SD | 5.28 ± 1.54 | 5.52 ± 2.4 | 0.553 |
| Body Mass Index , mean ± SD | 22.51 ± 2.50 | 23.17 ± 3.15 | 0.249 |

TRISDS = trans-intersphincteric double seton; I&D = incision and drainage; SD = standard deviation.

**Supplementary Table S2.** Comparison of wound conditions between TRISDS and I&D groups in per-protocol population

| Variable | TRISDS  (*n* = 50) | I&D  (*n* = 50) | *P* value |
| --- | --- | --- | --- |
| Wound exudate, median (IQR) |  |  |  |
| Postoperative 24 hours | 3.0 (2.0–3.0) | 2.5 (2.0–3.0) | 0.077 |
| Postoperative 1 week | 2.0 (1.0–2.25) | 1.5 (1.0–2.0) | 0.350 |
| Postoperative 2 weeks | 1.0 (0.0–1.0) | 1.0 (0.0–1.0) | 0.238 |
| Bud growth, median (IQR) |  |  |  |
| Postoperative 24 hours | 3.0 (2.0–3.0) | 3.0 (2.75–3.0) | 0.193 |
| Postoperative 1 week | 2.0 (1.0–2.0) | 2.0 (1.0–2.0) | 0.122 |
| Postoperative 2 weeks | 1.0 (0.0–1.25) | 1.0 (1.0–2.0) | 0.158 |
| Traumatic edema, median (IQR) |  |  |  |
| Postoperative 24 hours | 0.0 (0.0–1.0) | 0.5 (0.0–1.0) | 0.382 |
| Postoperative 1 week | 0.0 (0.0–1.0) | 1.0 (0.0–1.0) | 0.399 |
| Postoperative 2 months | 0.0 (0.0–1.0) | 0.0 (0.0–1.0) | 0.433 |

TRISDS = trans-intersphincteric double seton; I&D = incision and drainage; IQR = interquartile range.

**Supplementary Table S3.** Comparison of outcomes between TRISDS and I&D groups in per-protocol population

| Variable | TRISDS  (n=50) | I&D  (n=50) | *P* value |
| --- | --- | --- | --- |
| Pain score, mean ± SD |  |  |  |
| Postoperative 24 hours | 6.86 ± 1.04 | 7.50 ± 0.95 | 0.002* |
| Postoperative 1 week | 3.64 ± 1.15 | 3.38 ± 0.98 | 0.230 |
| Postoperative 2 weeks | 1.58 ± 0.90 | 1.36 ± 0.59 | 0.115 |
| Length of hospital stay,days,  mean ± SD | 4.06 ± 1.54 | 3.38 ± 1.43 | 0.024* |
| Treatment failure, n (%) |  |  |  |
| Fistula formation | 5 (10) | 18 (36) | 0.002* |
| Recurrence of abscesses | 2 (4) | 11 (22) | 0.007* |
| Total | 7 (14) | 29 (58) | <0.001* |
| Median Wexner score,  median (IQR) |  |  |  |
| Preoperative | 0.0 (0.0–1.0) | 0.0 (0.0–1.0) | 0.677 |
| Postoperative 2 weeks | 1.0 (1.0–2.25) | 1.0 (0.0–2.0) | 0.030* |
| Postoperative 2 months | 1.0 (0.0–1.0) | 1.0 (0.0–1.0) | 0.290 |

TRISDS = trans-intersphincteric double seton; I&D = incision and drainage; IQR = interquartile range; SD = standard deviation.

*: *P* < 0.05.
